# Aboriginal and non-Aboriginal sexually transmitted infections and blood borne virus notification rates in Western Australia: using linked data to improve estimates

**DOI:** 10.1186/1471-2458-13-404

**Published:** 2013-04-29

**Authors:** Rochelle E Watkins, Donna B Mak, Carolien M Giele, Sharon Clews

**Affiliations:** 1 Communicable Disease Control Directorate, Public Health Division, Health Department of Western Australia, Perth, Australia; 2 Telethon Institute for Child Health Research, Centre for Child Health Research, The University of Western Australia, Perth, Australia

## Abstract

**Background:**

National notification data for sexually transmitted infections (STIs) and blood borne viruses (BBVs) continue to have a high proportion of missing data on Indigenous status, potentially biasing estimates of notification rates by Aboriginality. We evaluated the use of data linkage to improve the accuracy of estimated notification rates for STIs and BBVs in Aboriginal and non-Aboriginal groups in Western Australia.

**Methods:**

STI and BBV case notifications in Western Australia received in 2010 were linked with administrative health data collections in Western Australia to obtain additional data on Indigenous status. STI and BBV notification rates based on the pre- and post-linkage data among Aboriginal and non-Aboriginal groups were compared.

**Results:**

Data linkage decreased the proportion of notifications with unknown Indigenous status by 74% from 10.2% to 2.7%. There was no significant difference in disease-specific age-adjusted notification rate ratio estimates based on pre-linkage data and post-linkage data for Aboriginal people compared with non-Aboriginal people.

**Conclusion:**

Our findings suggest that reported STI and BBV disease-specific age-adjusted notification rates for 2010 in Western Australia are unlikely to be significantly biased by excluding notifications with unknown Indigenous status. This finding is likely to be dependent on recent improvements in the reporting of Indigenous status in notification data in Western Australia. Cost-effective and systematic solutions, including the better use of existing data linkage resources, are required to facilitate continued improvement in the completeness of reporting and accuracy of estimates for notifiable STIs and BBVs in Australia by Aboriginality.

## Background

Substantial deficits persist in the reporting of Indigenous status for notifications of sexually transmitted infections (STIs) and blood borne viruses (BBVs) in Australia, with approximately half of nationally notified chlamydia and hepatitis C cases, and over one third of gonorrhoea cases missing data on Aboriginality in 2010 [1]. Consistent with the significantly greater burden of disease and socioeconomic disadvantage among Indigenous Australians generally [2,3], Indigenous Australians are overrepresented in national STI and BBV notification data [1,4].

Notifiable disease surveillance systems provide timely information for disease control policy and practice, and accurate estimation of disease notification rates by Aboriginality is critical to enable the effective evaluation of interventions to improve disease detection, treatment and prevention. The completeness of case notification data, including the completeness of information on Indigenous status, can influence the validity and usefulness of estimated notification rates derived from routinely collected disease surveillance data [5]. Improved Indigenous identification in communicable disease notification data is needed to provide a clearer understanding of the burden of communicable diseases in Australia, enable improved use of these data to address communicable diseases in Indigenous populations, and contribute to enhanced health and well-being among Indigenous people [6].

National disease notification data have shown little improvement in the completeness of data on Indigenous status over the previous 5 years. Only 50% of all national case notifications received by the National Notifiable Disease Surveillance System (NNDSS) in 2009 had known Indigenous status [4], reflecting an increase of less than 5% over 2004 levels [7]. Case notifications with unknown or incomplete data on Indigenous status are commonly excluded in the estimation of disease rates by Aboriginality, a strategy called complete case analysis. Complete case analysis is widely used in the presence of incomplete data, yet this strategy can bias estimates in ways that are difficult to predict unless the proportion of incomplete cases is small [8]. Estimates based on incomplete data are influenced by the amount of incomplete data, the factors that influence incompleteness, and the degree of similarity between complete cases and incomplete cases [8].

In addition to the influence of incomplete data, the misclassification of Aboriginality in disease notification data also has the potential to bias estimated notification rates among Indigenous and non-Indigenous populations. An analysis of STI and BBV notifications in Western Australia during 2004 found that high levels of incompleteness in the reporting of Indigenous status can contribute to overestimation of the risk associated with Aboriginality for some disease notifications [5]. However, the influence of misclassification was only examined among notifications with unknown Aboriginality, and there has been little investigation of the extent of misclassification in notifiable disease data.

Data from states and territories where a high proportion of cases have unknown Indigenous status are routinely excluded from national estimates due to the potential to underestimate the true prevalence of these infections among Indigenous Australians [1,4]. This exclusion of data introduces uncertainty in the estimation of STI and BBV notification rates, particularly when the proportion of incomplete cases is high. Valid estimates depend on high data completeness [9], and a low level of misclassification.

Trends suggest that the identification of Indigenous status for STI and BBV notifications in Western Australia is improving [10]. The accuracy of data on Indigenous status in other Western Australian administrative health databases has also improved among more recently collected data [11]. These improvements in data quality are likely to enable more accurate estimates of STI and BBV notification rates. However, data linkage remains rarely used to improve the quality of infectious disease surveillance data, and there has been no investigation of the impact of misclassification due to inconsistency in the reporting of Aboriginality on estimated STI and BBV notification rates. The aim of this study was to evaluate the use of data linkage to improve the accuracy of estimated Aboriginal and non-Aboriginal notification rates for STIs and BBVs in Western Australia using notification data received during 2010, and to identify implications for the routine analysis of notification data by Aboriginality.

## Methods

### Data sources and linkage

All notified cases of STIs (chlamydia, gonorrhoea, syphilis and donovanosis) and BBVs (hepatitis B and hepatitis C) in Western Australia with a case report date between the 1st January 2010 and 31st December 2010 were extracted from the Western Australian Notifiable Infectious Diseases Database (notification data) on 27th September 2011. This analysis excludes HIV cases which are notified separately and have complete data on Aboriginality. For each individual who was notified with a STI or BBV in 2010, data on Aboriginality were obtained from five administrative health data collections via the Western Australian Data Linkage System (WADLS). The WADLS is an established system that enables the creation and maintenance of links between administrative health data collections [12]. Notification data are routinely linked using the WADLS. This process was established to facilitate surveillance of hospitalisations due to notifiable infectious diseases in the event of an outbreak, and is not used to improve the quality or completeness of infectious disease notification data.

For this non-routine analysis, the following data were extracted for each notification to enable data linkage: notification identification number, first name, last name, sex, date of birth and residential address. Data linkage was based on probabilistic matching of records using multiple data fields, and was performed by experienced staff at the Data Linkage Branch. The standard methods used to link records, which includes manual review of links which do not meet predefined matching criteria, are described in more detail elsewhere [13]. Linked health records were provided using encrypted individual root numbers to protect individual privacy. This study was approved by the Western Australian Aboriginal Health Information and Ethics Committee and the Department of Health Western Australia Human Research Ethics Committee.

Data linkage obtained all available records from the following five health data collections: Hospital Morbidity Data Collection, Emergency Department Data Collection, Mental Health Information System, Midwives Notification System, and the Mortality Register. The population coverage of these data collections are congruent with the Western Australian Notifiable Infectious Diseases Database, with all providing data on the Western Australian population. For each linked health record identified in the five external databases, data on Aboriginality were obtained. Records from these external data collections are referred to as ‘external data’ and were analysed collectively. The combination of these external data and the Western Australian Notifiable Infectious Diseases Database are referred to as the ‘linked data’.

### Assignment of Aboriginality

The coding of Aboriginality varied in the five external data collections, with only the Emergency Department Data Collection, Mental Health Information System and Mortality Register including a specific code for unknown Aboriginality. The external data were recoded to describe reported Aboriginality as either Aboriginal (Aboriginal or Torres Strait Islander), non-Aboriginal (non-Aboriginal or Torres Strait Islander, or other) or unknown.

Two indicators of Aboriginality were derived using the linked data to examine the influence of the inconsistent classification of Aboriginality on estimated notification rates and rate ratios. These indicators were selected based on the assumption that, in administrative health data, Indigenous status is less likely to be coded accurately among Aboriginal people compared with non-Aboriginal people. A study of 10,106 inpatients in Western Australia found that in metropolitan hospitals, Indigenous status was accurately classified among 78.3% of patients who identified as Aboriginal, and 99.6% of patients who identified as non-Aboriginal [14]. Discrepancies in reported Indigenous status were also identified among an undisclosed proportion of the 319 patients who were interviewed on more than one occasion, highlighting the challenges in identifying a gold standard for comparison. Similarly, a study of 993 self-identified urban Aboriginal people living in Perth found that only 40% of individuals were correctly identified as an Aboriginal person in every Hospital Morbidity Data Collection record between 1980 and 2006, and that 10% of individuals were not coded as an Aboriginal person in any admission [11].

As such, the following two comparisons were used to calculate disease-specific age-standardised notification rates and rate ratios to reflect the likely under-identification of Aboriginal people in the linked data:

1) Ever Aboriginal (ever identified as Aboriginal in any notification or external data record) compared with never Aboriginal (never identified as Aboriginal in all notification and external data records where Aboriginality was known – i.e. consistently identified as non-Aboriginal), and

2) ≥ 25% Aboriginal (identified as Aboriginal in ≥ 25% of all notification and external data records where Aboriginality was known) compared with < 25% Aboriginal (identified as Aboriginal in < 25% of all notification and external data records where Aboriginality was known – i.e. > 75% non-Aboriginal)

Our use of the ≥ 25% indicator was selected as the midpoint between the ever indicator (> 0%) and an indicator based on the majority of records. Notifications that were consistently identified as unknown in the linked data were excluded from the analysis, and rate ratios were based on classification of all notifications with known Aboriginality following data linkage into two mutually exclusive groups.

### Data analysis

Descriptive statistics were used to summarise the consistency in the coding of Aboriginality in the linked data. The Chi-square test of independence was used to examine the association between the presence of unknown Aboriginality in the notification data and the following case characteristics: age group (0–24, 25+ years), sex, region of residence (metropolitan, non-metropolitan), disease, and the identification of Aboriginality following data linkage.

Age-standardised disease notification rates per 100,000 population were calculated using the pre-linkage notification data and the two indicators of Aboriginality derived from the linked data. Disease notification rates were age-standardised using the direct method, with the total estimated resident population in Australia on the 30th June 2001 used as the reference population [15]. Estimates of the Western Australian resident population by Aboriginality and 5-year age categories for 2010 used to calculate age-standardised disease notification rates were obtained from the Epidemiology Branch of the Department of Health Western Australia via the Rates Calculator [16], and are based on the 2006 Census [17].

Confidence intervals for age-standardised notification rates were calculated using estimates of variance based on the Poisson distribution [18]. Standardised rate ratios (RRs) of notification rates by Aboriginality were also calculated for each estimation method. To quantify the differences in age-standardised notification rates by Aboriginality, Poisson regression [19] was used to calculate age-adjusted notification rate ratios by Aboriginality and their 95% confidence intervals. Due to the sparseness of the data, regression analysis used standard 5-year age categories from 0–4 years to 75 or more years, and for syphilis the lower four age categories were also collapsed due to the lack of cases in individuals aged less than 15 years. SPSS version 19 (SPSS Inc., 2010) was used to analyse the data.

## Results

A total of 13,696 notifications for STIs and BBVs (excluding HIV) were received by the Department of Health Western Australia during 2010 and extracted for linkage. Records for 13,694 of the 13,696 notifications were successfully linked, and indicated that 12,597 individuals had been notified with a STI and or BBV in 2010. Data for the two notifications that were unable to be linked to the external data were retained in the analysis as 2 additional individuals (i.e. were considered independent of the individuals who were able to be linked to the external data).

A total of 182,156 health records were retrieved from the external data collections and linked to 10,566 of the 12,599 individuals notified with a STI and or BBV in 2010. A median of seven (range 1–2370) external data records were identified for the 10,566 individuals with linked external data, and 1,076 of these individuals (10.2%) had only one linked record in the external data.

### Identification of Aboriginality

The proportion of notifications with unknown Aboriginality decreased from 10.2% prior to data linkage to 2.7% following data linkage (Table 1). Chlamydia had the highest proportion cases with unknown Aboriginality prior to data linkage, and hepatitis B had the highest proportion cases with unknown Aboriginality following data linkage.

**Table 1:** Summary of STI and BBV notifications received in 2010 with unknown Aboriginality by disease based on notification data, notification data internally linked by individual, and the linked notification and external data

**Disease**	**Notification data pre-linkage: Unknown Aboriginality**	**Notification data internally linked:**^**† **^**Unknown Aboriginality**	**Linked notification and external data: Unknown Aboriginality**^**‡**^
	**n (%)**	**n (%)**	**n (%)**
Chlamydia	1276 (12.5)	1188 (11.6)	285 (2.8)
Gonorrhoea	4 (0.3)	3 (0.2)	2 (0.1)
Syphilis infectious	0 (0)	0 (0)	0 (0)
Syphilis non-infectious	0 (0)	0 (0)	0 (0)
Donovanosis	0 (0)	0 (0)	0 (0)
Hepatitis B	64 (7.9)	60 (7.4)	46 (5.7)
Hepatitis C	52 (4.8)	52 (4.8)	32 (3.0)
**Total (n = 13696)**	**1396 (10.2)**	**1303 (9.5)**	**365 (2.7)**

^†^internal linkage based on identification of individuals who had more than one case notification in 2010.

^‡^unknown in both data sources (notification and external data).

The association between having unknown Aboriginality in the notification data and sociodemographic characteristics and disease is summarised in Table 2. Notifications were significantly more likely to have missing data on Aboriginality prior to data linkage if the case was ≥ 25 years of age, notified in the metropolitan area, notified with chlamydia or hepatitis B, or had never been identified as Aboriginal in the linked data.

**Table 2:** Association between missing data on Aboriginality in notification data prior to data linkage and sociodemographic characteristics and disease

**Characteristic**	**Total notifications (n = 13696) n (%)**	**Unknown Aboriginality n (%)**	***χ***^**2 **^**p**	**Unknown Aboriginality odds ratio (95%CI)**
Sex			0.95	1.00 (0.90-1.10)
Male	6544 (47.8)	666 (10.2)		
Female	7152 (52.2)	730 (10.2)		
Age			0.01	0.88 (0.80-0.97)
<25 years	7774 (56.8)	748 (9.6)		
25+ years	5922 (43.2)	648 (10.9)		
Region of residence^†^			0.006	0.85 (0.76-0.96)
Non-metropolitan	4137 (30.2)	370 (8.9)		
Metropolitan	9438 (68.9)	989 (10.5)		
Disease			<0.001	
Chlamydia	10248 (74.8)	1276 (12.5)		
Gonorrhoea	1405 (10.3)	4 (0.3)		
Syphilis infectious	83 (0.6)	0 (0)		
Syphilis non-infectious	68 (0.5)	0 (0)		
Hepatitis B	811 (5.9)	64 (7.9)		
Hepatitis C	1080 (7.9)	52 (4.8)		
Ever Aboriginal in linked data				
No	10810 (78.9)	1328 (12.3)	<0.001	5.21 (4.10-6.63)
Yes	2886 (21.1)	68 (2.4)		

^†^Excludes 121 notifications with no specified Western Australian region of residence.

Individuals who were ever identified as Aboriginal in the linked data were significantly more likely to have a greater number of linked records (median = 20) when compared with participants who were never identified as Aboriginal (median = 5; Z = -52.2, p < 0.001). Among the notifications ever identified as Aboriginal in the linked data, 63.2% were identified consistently as Aboriginal in all records with known Aboriginality. Among the notifications ever identified as non-Aboriginal in the linked data (n = 11,506), 90.8% were identified consistently as non-Aboriginal in all records with known Aboriginality.

### Disease notification rates

The Aboriginality of STI and BBV notifications prior to and following data linkage is shown by estimation method in Table 3. Differences between the pre-linkage and post-linkage proportion of disease notifications according to Aboriginality were generally small. Compared with pre-linkage estimates, an increased proportion of notifications were classified as Aboriginal using both post-linkage definitions of Aboriginality for all diseases apart from non-infectious syphilis and donovanosis. Non-infectious syphilis and donovanosis were the least frequently notified diseases and had no missing data on Aboriginality prior to data linkage. Only chlamydia and hepatitis B showed a consistent increase in the proportion of notifications classified as non-Aboriginal based on both post-linkage definitions of non-Aboriginality compared with pre-linkage estimates; however, the magnitude of increase for hepatitis B was small.

**Table 3:** Comparison of the Aboriginality of STI and BBV notifications received in 2010 prior to and following data linkage by estimation method

	**Aboriginal**			**Non-Aboriginal**		
**Disease**	**Pre-linkage**	**Post-linkage ever**	**Post-linkage ≥ 25%**	**Pre-linkage**	**Post-linkage consistent**	**Post-linkage > 75%**
	**n (%)**	**n (%)**	**n (%)**	**n (%)**	**n (%)**	**n (%)**
Chlamydia	1571 (15.3)	1772 (17.3)	1651 (16.1)	7401 (72.2)	8191 (79.9)	8312 (81.1)
Gonorrhoea	842 (59.9)	863 (61.4)	850 (60.5)	559 (39.8)	540 (38.4)	553 (39.4)
Syphilis infectious	19 (22.9)	21 (25.3)	20 (24.1)	64 (77.1)	62 (74.7)	63 (75.9)
Syphilis noninfectious	16 (23.5)	16 (23.5)	16 (23.5)	52 (76.5)	52 (76.5)	52 (76.5)
Donovanosis	1 (100)	1 (100)	1 (100)	0 (0)	0(0)	0 (0)
Hepatitis B	44 (5.4)	47 (5.8)	46 (5.7)	703 (86.7)	718 (88.5)	719 (88.7)
Hepatitis C	135 (12.5)	166 (15.4)	138 (12.8)	893 (82.7)	882 (81.7)	910 (84.3)
**Total (n = 13696)**	**2628 (19.2)**	**2886 (21.1)**	**2722 (19.9)**	**9672 (70.6)**	**10445 (76.3)**	**10609 (77.5)**

Estimated disease rates and rate ratios following data linkage showed few differences with pre-linkage estimates (Table 4). For chlamydia, which had the highest proportion of notifications with unknown Aboriginality prior to linkage, estimated notification rates increased significantly both among Aboriginal and non-Aboriginal people based on the ‘ever Aboriginal’, ‘consistent non-Aboriginal’ and ‘> 75% non-Aboriginal’ definitions. Rates of STIs and BBVs among Aboriginal people remained significantly higher than among non-Aboriginal people, with the confidence interval of all disease-specific rate ratios excluding 1. For all STIs and BBVs examined, estimated post-linkage age-adjusted notification rate ratios were not significantly different when compared with pre-linkage estimates, indicating that record linkage did not significantly alter the relative proportion of disease notifications occurring among Aboriginal and non-Aboriginal people using either estimation method.

**Table 4:** Comparison of age-standardised notification rates (per 100,000 population) and standardised rate ratios (RR) of STI and BBV notifications received in 2010 by Aboriginality prior to and following linkage by estimation method

**Disease**	**Pre-linkage Aboriginal**	**Pre-linkage Non-Aboriginal**	**Pre-linkage RR**^**†**^	**Ever Aboriginal**	**Consistent non-Aboriginal**	**Ever-Consistent RR**^**†**^	**≥ 25% Aboriginal**	**> 75% non-Aboriginal**	**25%-75% RR**^**†**^
	**rate (95%CI)**	**rate (95%CI)**	**(95% CI)**	**rate (95%CI)**	**rate (95%CI)**	**(95% CI)**	**rate (95%CI)**	**rate (95%CI)**	**(95% CI)**
Chlamydia	1573.7	325.2	5.0	1778.6	360.1	5.1	1657.1	365.5	4.7
	(1493.5-1653.9)	(317.7-332.6)	(4.7-5.2)	(1693.3-1863.9)	(352.3-367.9)	(4.8-5.3)	(1574.7-1739.5)	(357.6-373.4)	(4.4-4.9)
Gonorrhoea	895.8	24.9	38.4	920.4	24.0	40.8	906.3	24.6	39.2
	(833.2-958.5)	(22.8-27.0)	(34.5-42.8)	(856.8-983.9)	(22.0-26.0)	(36.6-45.5)	(843.2-969.4)	(22.5-26.7)	(35.2-43.7)
Syphilis infectious	24.2	2.9	9.0	27.5	2.8	10.4	25.8	2.8	9.7
	(12.9-35.4)	(2.2-3.6)	(5.4-15.1)	(15.3-39.7)	(2.1-3.5)	(6.3-17.1)	(14.1-37.6)	(2.1-3.5)	(5.8-16.1)
Syphilis noninfectious^‡^	39.0	2.3	11.7	-	-	-	-	-	-
	(15.4-62.6)	(1.7-3.0)	(6.6-20.7)	-	-	-	-	-	-
Hepatitis B	98.6	31.8	2.0	108.0	32.5	2.1	105.9	32.5	2.0
	(61.8-135.5)	(29.4-34.1)	(1.5-2.7)	(69.0-147.1)	(30.1-34.8)	(1.5-2.8)	(67.1-144.8)	(30.1-34.9)	(1.5-2.7)
Hepatitis C	187.5	40.2	5.1	232.0	39.8	6.3	193.3	41.0	5.1
	(154.9-220.1)	(37.6-42.9)	(4.2-6.1)	(195.6-268.4)	(37.1-42.4)	(5.3-7.5)	(160.2-226.5)	(38.3-43.7)	(4.2-6.1)

^†^Estimates based on Poisson regression model adjusted for age.

^‡^Post-linkage estimates are identical to pre-linkage estimates and have been omitted from the table.

## Discussion

Compared with pre-linkage notification rate ratios, we found no significant difference in age-adjusted STI and BBV notification rate ratios by Aboriginality in Western Australia following data linkage. Complete and accurate data on Aboriginality are required for the valid estimation of STI and BBV notification rates, and despite investigating the influence of both incomplete data and the inconsistent classification of Aboriginality on Aboriginal to non-Aboriginal notification rate ratios in 2010, variability in the post-linkage estimates was small. These findings support the validity of estimating disease-specific notification rate ratios in 2010 based on the exclusion of cases with unknown Aboriginality, and contrast with an analysis of 2004 data which found that the exclusion of cases with unknown Aboriginality prior to linkage overestimated disease-specific notification rate ratios for some diseases [5].

A review of the findings in 2004 reveals that data linkage identified data on Aboriginality for a similar proportion of notifications with unknown Aboriginality prior to linkage in 2004 (73.5%) and 2010 (73.9%), and that only a small proportion of notifications with unknown Aboriginality prior to data linkage were ever identified as Aboriginal following data linkage in both 2004 (5.8%) and 2010 (4.9%). However, the proportion of STI and BBV notifications with unknown Aboriginality prior to data linkage decreased approximately 60% from 26% in 2004 to 10% in 2010, and there was a large decrease in age-adjusted notification rate ratios by Aboriginality between 2004 and 2010 for all STIs and BBVs apart from hepatitis C. Improved accuracy of identification of Indigenous status in more recent data has also been found in a study of the Western Australian Hospital Morbidity Data Collection [11], suggesting that recent improvements in data quality are not limited to notifiable disease data.

Improved completeness of Indigenous status in the notification data can be attributed to better reporting by medical practitioners and greater awareness of the importance of high levels of data completeness among staff in public health units who receive and manage the data. STI and BBV case notifications with missing data on Indigenous status are followed-up by public health unit staff who use several strategies to improve the completeness of notifications, including contacting the notifying medical practitioner; using their knowledge of the local community, particularly in regional areas where the local community is small; and using information from previous notifications and other administrative health data collections, including the public hospital database ‘The Open Patient Administration System’ (TOPAS).

There is currently no mechanism in the Western Australian Notifiable Infectious Diseases Database to identify whether notification data on Indigenous status were obtained from the original notification or during follow-up, and only a detailed audit of the original paper notification records could retrospectively identify the contribution of follow-up processes to reporting completeness. However, cases which are only laboratory notified (i.e. no notification is received from a medical practitioner) do not include information on Indigenous status, and the development of strategies to establish reporting of Indigenous status for these cases is required. Anecdotal information indicates that follow-up processes conducted to improve the completeness of data on Aboriginality are essential to maintain a high level of identification of Indigenous status in the STI and BBV notification data. Ensuring that the contribution of follow-up processes to reporting completeness are identifiable could enable both the evaluation of strategies developed to improve reporting completeness and the development of processes to maintain the high levels of reporting achieved in 2010.

The completeness of Aboriginality in the notification data varied by disease according to previously reported patterns [5], and the low proportion of syphilis and gonorrhoea notifications with unknown Aboriginality prior to data linkage reflect the use of enhanced surveillance processes for these diseases in Western Australia [20]. STI and BBV rates and rate ratios can reveal changes in endemic and epidemic activity as well as changes in disease detection and control efforts. The largest decline in the estimated age-adjusted rate ratio between 2004 and 2010 was observed for infectious syphilis, which reflects a continuation of the documented decline in notifications among Aboriginal people and an increase in notifications among non-Aboriginal people [21].

In the absence of a gold standard indicator of Indigenous status, the interpretation of these findings depends on assumptions about the accuracy of information on Aboriginality in the linked data and the validity of methods used to define Aboriginality. Data linkage can be used to improve the quality of Indigenous data [22], but has a limited ability to identify the misclassification of Indigenous status in administrative health data. Research suggests that administrative data collections are likely to under-identify Aboriginal people [11], and we found that unknown Indigenous status in STI and BBV notification data is significantly associated with sociodemographic factors and disease, with unknown Aboriginality more likely to occur among notifications identified as non-Aboriginal following data linkage.

Little data are available to allow estimation of the extent of misclassification of Aboriginality in infectious disease notification data, and identification of the most appropriate definition of Aboriginality when multiple inconsistent indicators exist. Due to inconsistency in the coding of, and uncertainty associated with the validity of indicators of Aboriginality in the linked data, two different criteria were used to examine the impact of classification method on the disease-specific standardised rates and rate ratios. Alongside research demonstrating poorer identification of Aboriginal people in administrative health databases and more accurate coding of Indigenous status among non-Aboriginal people [11,14], our finding of poorer consistency in reporting among notifications ever identified as Aboriginal supports the use of less rigorous criteria to identify notifications among Aboriginal people based on multiple linked records.

Although disease-specific notification rates were higher than pre-linkage estimates when the ever-Aboriginal definition of Aboriginality was used, significant differences in disease rates were found only for Chlamydia, which had the highest proportion of records with unknown Aboriginality prior to linkage. The use of the ever Aboriginal indicator had little effect on the estimated disease-specific notification rate ratios by Aboriginality despite the potential vulnerability of this indicator to overestimation based on the presence of a single misclassification. Given the likely underestimation of Aboriginality in administrative health data, and the similarity between the three notification rate ratio estimates in this analysis, our findings suggest that indicators based on ever Aboriginal and never Aboriginal may provide an appropriate basis for the calculation of STI and BBV notification rate ratios until an indicator with improved validity is available.

A range of factors have been found to influence the collection and recording of indigenous status in health records, including a lack of awareness and training among staff, staff reluctance to ask the question, staff perceptions that Indigenous Australians do not want to disclose their status; a lack of privacy when answering the question; refusal to answer the question; and little validation of data or follow-up of missing information [10]. Despite the development of best practice guidelines to promote the collection of correct and consistent information on Aboriginality [10], information on Indigenous status is not always consistently sought by health service providers or consistently provided by health service consumers, and self-identification may vary in different contexts. Guidelines recommend the need for staff training; mechanisms for quality assurance and validation, including business rules for checking indigenous status against other data items; and the need to ensure consistency between identifications when there are multiple sources of data [10].

Limitations of this analysis include the lack of a gold standard for comparison and the large variation observed in the number of linked records identified for each individual. The presence of inconsistent classification of Aboriginality examined in this analysis provides only a limited indicator of misclassification in the linked data. The validity of notification rate ratios can also be affected by inaccuracies in the estimated population denominators, the failure to notify, and the under-diagnosis of STIs and BBVs, particularly in rural and remote areas where there is poorer access to health services. In addition, summary rate ratios can mask significant variation in age-specific rate ratios, and some estimates were based on small case numbers.

A high level of completeness of data on Indigenous status in STI and BBV notifications is critical for the accurate estimations of disease rates by Aboriginality. Given the sustained poor reporting of Indigenous status among STI and BBV notifications nationally, there is a need to consider the use of additional strategies to improve the completeness of reporting. Strategies could include better utilisation of existing data linkage resources and the development of indicators that can be used to enable more complete and accurate identification of Aboriginality in routine health surveillance analyses.

The routine linkage of Western Australian notification data with the WADLS could be utilised to improve completeness of data on Indigenous status through the internal linkage of notification data from multiple years by individual. Internal data linkage by individual requires few additional resources, although is likely to provide only modest improvements in data completeness compared with external linkage with other data collections. External linkage is currently more resource intensive and unlikely to be cost effective when there is a low proportion of notifications with unknown Aboriginality. However, routine external linkage may be an effective strategy for other notifiable diseases or in other jurisdictions where the completeness of Indigenous status is low.

## Conclusions

We found improved reporting of Indigenous status in STI and BBV notification data in Western Australia in 2010 compared with 2004, and that STI and BBV disease-specific age-adjusted notification rates for 2010 are unlikely to be significantly biased by excluding notifications with unknown Indigenous status. Better use of existing data linkage resources, including the development of a standard systematic approach to the identification and reporting of health indicators by Aboriginality as has been identified by Draper and co-workers [23], could contribute to continued improvement in the completeness of reporting and accuracy of estimates for STIs and BBVs in Australia by Aboriginality. The availability of a standard indicator with established validity would provide an efficient and cost-effective means to validate and improve the quality of multiple health indicators.

## Competing interests

The authors declare that they have no competing interests.

## Authors’ contributions

All authors were involved in designing the study. DBM supervised the study, CMG extracted the notification data for the study, REW analysed the data and drafted the manuscript, and all authors critically reviewed the manuscript.

## Pre-publication history

The pre-publication history for this paper can be accessed here:

http://www.biomedcentral.com/1471-2458/13/404/prepub
